# Unilateral Cytomegalovirus Retinitis in a Child With Acute Lymphoblastic Leukemia While on Maintenance Chemotherapy

**DOI:** 10.7759/cureus.15246

**Published:** 2021-05-26

**Authors:** Rahaf A Mandura, Karim Talat, Wasil Jastaniah

**Affiliations:** 1 Department of Ophthalmology, King Abdulaziz University, Jeddah, SAU; 2 Department of Ophthalmology - Vitreoretinal Surgery, King Abdulaziz Medical City, Jeddah, SAU; 3 Department of Pediatrics, Princess Noorah Oncology Center, King Abdulaziz Medical City, Ministry of National Guard Health Affairs, Jeddah, SAU

**Keywords:** cytomegalovirus, cytomegalovirus retinitis, acute lymphoblastic leukemia, pediatric, all, hematology

## Abstract

Cytomegalovirus retinitis (CMVR) commonly affects immunocompromised individuals, including acquired immunodeficiency syndrome (AIDS), post-organ transplant recipients and allogeneic stem cell transplant recipients. CMVR occurring in the acute lymphoblastic leukemia (ALL) maintenance phase of chemotherapy is rare and has been described in the literature as isolated case reports or case series. We report a case of unilateral CMVR in a pediatric patient during maintenance phase therapy for ALL. A 14-year-old boy known case of T-cell ALL with CNS2a status, was treated according to the Children’s Oncology Group (COG) AALL0434 protocol. Induction therapy consisted of the standard high-risk four drugs, in addition to intrathecal methotrexate. At week 166 of maintenance therapy, the child presented with painless progressive loss of vision in the right eye for one week. The best-corrected visual acuity (BCVA) of the right eye was 6/36 and the left eye was 6/6. Dilated fundus examination of the right eye showed multiple large yellow-white cloudy chorioretinal lesions with areas of intraretinal hemorrhages in the macula, and overlaying focal vitritis. Optical coherence tomography (OCT) of the right eye showed macular edema and mild subretinal fluid. Cytomegalovirus polymerase chain reaction of the blood was detected with high quantitative value. A diagnosis of CMVR was made and an induction doses of intravenous ganciclovir was followed by maintenance doses of oral valganciclovir. Our case suggests that pediatric patients with ALL in the maintenance phase are considered immunocompromised and that physicians should be aware of CMVR incidence in such group. Early diagnosis and prompt treatment are important to preserve vision and prevent future visual morbidity.

## Introduction

Cytomegalovirus (CMV) is a type 5 herpes virus causing an asymptomatic or minimally symptomatic illness in immunocompetent individuals [1]. Furthermore, it is considered a common opportunistic intraocular infection in acquired immune deficiency syndrome (AIDS) secondary to the depressed cell-mediated immunity when CD4 counts decline to less than 100/μL [2]. It can also occur in patients who received a solid organ or hematopoietic stem cell transplant [1]. Cytomegalovirus retinitis (CMVR) due to CMV infection is very destructive and can cause full-thickness retinal inflammation, hemorrhage, and necrosis which can eventually result in serious complications such as retinal detachment and vision loss [3]. CMVR starts by affecting the vascular endothelial cells. Later, it affects the retinal pigment epithelium. After such manifestations occur, the virus' final cytopathic effect is subsequent retinal necrosis [4]. Acute lymphoblastic leukemia (ALL) patients undergoing maintenance chemotherapy rarely develop CMVR and are usually described as isolated case reports or case series in the literature [5-9]. We describe a case of unilateral CMVR in a pediatric patient during his maintenance phase therapy for ALL.

## Case presentation

**Figure 1:**
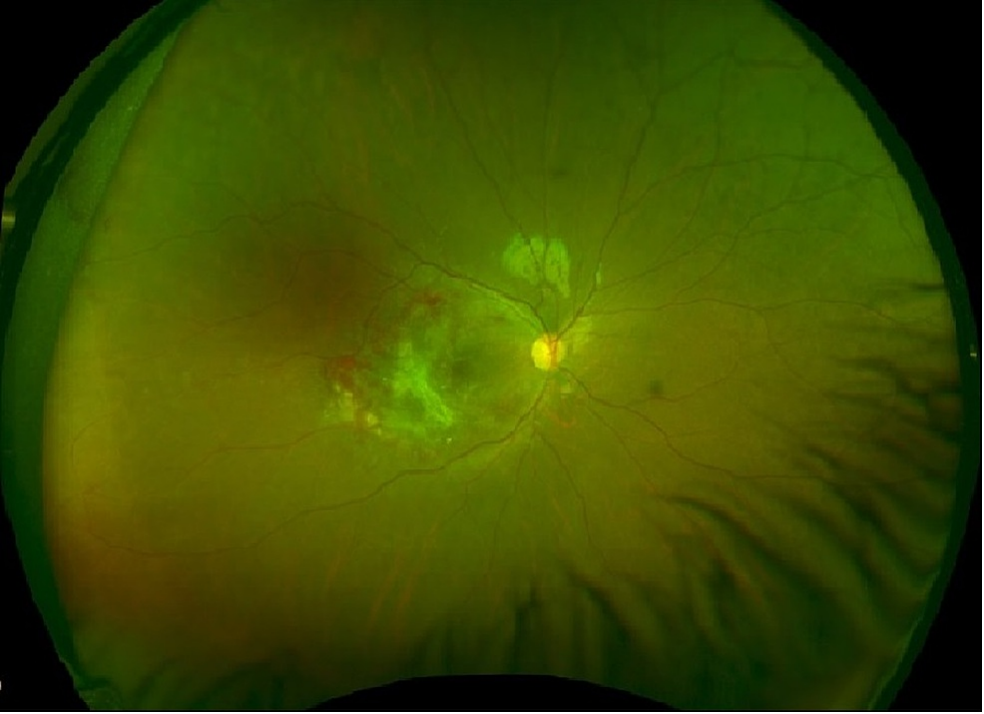
Fundus photo of the right eye showing CMV retinitis with retinal necrosis, intraretinal hemorrhages in the macula area, and focal overlying vitritis. CMV: cytomegalovirus.

**Figure 2:**
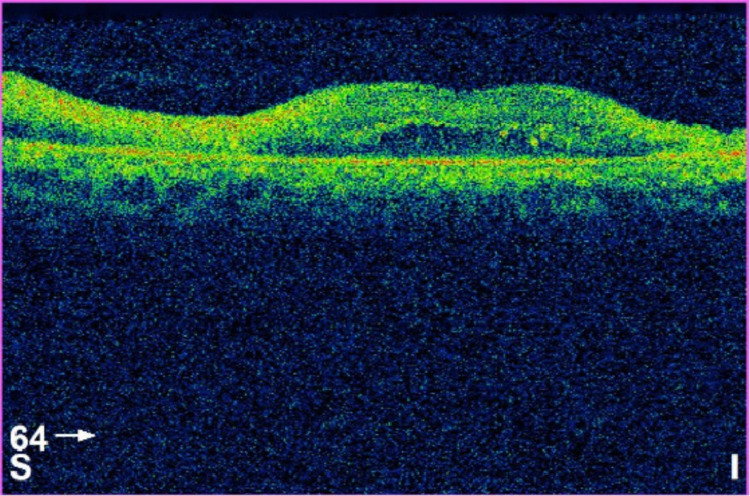
Optical coherence tomography of the right eye showing macular edema and subretinal fluid.

A 14-year-old boy who is a known case of T-cell acute ALL with CNS2a status presented with painless progressive loss of vision in the right eye for one week. Regarding his ALL history, he initially presented with leukocytosis of 572 x 10^9^/L and flow cytometry confirmed T-cell immunophenotype. He had no clinical symptoms or signs of neurological involvement and his vision was intact. However, he had evidence of blasts with low white blood cells on cyto-spin in his cerebrospinal fluid; thus, his central nervous system (CNS) involvement was categorized as CNS2a. Leukemia cytogenetics showed 46XY, del(6)(q21q23). He was treated according to the Children’s Oncology Group (COG) AALL0434 protocol [10]. Induction therapy consisted of the high-risk four-drug induction and intrathecal therapy. In addition, two extra intrathecal methotrexate doses were given during induction due to his CNS2a status. End-of-induction minimal residual disease (MRD) showed 6% bone marrow blasts and at the end-of-consolidation, his MRD was negative with <0.01% blasts. After completing the intensive phase therapy, he was started on maintenance therapy, which consisted of 12-week cycles of monthly vincristine, dexamethasone pulse therapy, daily oral 6-mercaptopurine and weekly oral methotrexate [10]. He tolerated therapy relatively well until week 166 of maintenance therapy when a painless progressive loss of vision in the right eye started. Prior ocular history was unremarkable with no previous ocular disease while his past medical history was significant for varicella-zoster reactivation at the left thoracic dermatome during week 154 of maintenance therapy and was treated with intravenous acyclovir for 10 days with complete resolution. On ocular exam, the best-corrected visual acuity (BCVA) of the right eye was 6/36 and the left eye was 6/6. Intraocular pressure measured by tonopen was 17 mmHg in the right eye and 15 mmHg in the left eye. Anterior segment examination was normal and anterior chambers were quiet in both eyes without evidence of keratic precipitates or anterior uveitis. Dilated fundus examination of the right eye showed multiple large yellow-white cloudy inflammatory chorioretinal lesions with granular appearance and active borders, the largest lesion measuring around three disc diameters which was located in the macular area extending to the center of the fovea centering around the major retinal arterial vasculature in the posterior pole and surrounding the optic disc. It was associated with significant full-thickness retinal necrosis, intraretinal hemorrhages, perivascular sheathing (perivasculitis) along the arcades, cotton wool spots (soft exudates) and mild focal vitritis overlying the whitish active lesions. No associated retinal breaks or rhegmatogenous retinal detachment were noted (Figure 1). Optical coherence tomography (OCT) of the right eye showed the necrotizing retina appeared swollen as compared to the surrounding areas with the presence of macular edema, mild subretinal fluid accumulation, disturbance of foveal architecture, hyperreflective intraretinal deposits and clumps of hyperreflective material in the posterior vitreous consistent with vitreous cells and debris (Figure 2) while the left eye was normal.

Blood test for CMV polymerase chain reaction (PCR) was performed, and CMV DNA was detected with the highest quantitative level of 409,000 copies/ml and a CD4 count of 2.5 cell/mm. The patient was diagnosed with probable right eye CMVR with no evidence of any other organ involvement based on the clinical and laboratory findings. The maintenance chemotherapy was discontinued, and anti-CMV therapy was started in the form of high induction doses of daily intravenous infusion of ganciclovir 10 mg/kg for four weeks with weekly CMV PCR test. After the patient showed good response from the systemic therapy, no further intravitreal therapy was warranted. On examination, BCVA of the right eye improved to 6/30, and the left eye was 6/6. Dilated fundus examination showed improvement with some resolution of the active retinitis and retinal hemorrhages. CMV PCR was repeated and showed a decreased quantitative level of 8,814 copies/ml.

On the second week's visit, BCVA of the right eye reached 6/9, and dilated fundus examination showed marked improvement and reduced size of retinitis lesions. Also, there was a considerable replacement of the retinal necrosis with thin whitish fibrous membrane and glial sheets and resolution of the focal vitritis with no sign of disease activity. CMV PCR dropped down to 622 copies/ml and OCT showed clear macula with complete resolution of subretinal fluid. On the subsequent days, the patient was kept on the maintenance doses of oral valganciclovir 900 mg twice daily, which was continued for eight months until complete resolution of symptoms was achieved along with three consecutive negative readings of CMV PCR. The patient resumed and completed chemotherapy therapy a few months later and has been in clinical remission for two years.

## Discussion

CMV viremia occurs in 13.6% of patients with lymphoid malignancies who did not receive stem cell transplantation [11]. CMVR is reported more frequently in children with AIDS than in other immunosuppressive conditions [12]. CMVR is characterized by spreading hematogenously to the retina, which happens after the systemic reactivation of latent infection [13]. An experienced ophthalmologist establishes the diagnosis of CMRV based on typical retina changes. Ocular pathophysiology of CMVR appears as full-thickness necrotizing retinitis. Furthermore, it appears as a fluffy, yellow-white retinal lesions, while intraretinal hemorrhage with little inflammation to the vitreous is an unpredictable sign found in the disease [14]. ALL patients developing CMVR happens mostly during maintenance chemotherapy. This maintenance phase is the most common period in the whole pediatric lymphoblastic group to develop CMV infection [15]. This finding could be due to the immunosuppressive chemotherapeutic agents used in this phase. It is hypothesized that the addition of dexamethasone and vincristine to methotrexate and 6-mercaptopurine may increase the risk of CMVR substantially [7]. The prevalence of CMV reactivation during the maintenance phase is often high and is caused by these drugs due to the development of lymphopenia [7].

We have treated our patient with intravenous ganciclovir for four weeks, followed by oral valganciclovir. The treatment was well tolerated by the patient without adverse effects and showed a good response resulting in a successful resolution of the CMVR, consistent with the literature [5-9]. The disadvantage of intravenous ganciclovir includes decreased bioavailability in ocular tissues in comparison to intravitreal ganciclovir and a significant relapse rate [12]. However, our patient was followed up for two years and no recurrence has been reported. In addition, systemic ganciclovir has the advantage of limiting the spread of CMV infection to the other eye and we believe that this prevention would not be possible if we considered intravitreal ganciclovir as a sole treatment.

## Conclusions

CMVR is a tremendous visual threat in immunocompromised patients. Chemotherapy-related immunosuppression in pediatric patients with ALL happens in the maintenance phase of chemotherapy with chances of developing CMVR, making it important to have such a diagnosis in mind when seeing patients with ALL. A careful and vigilant approach to such cases is crucial as early diagnosis and prompt treatment are important to preserve vision and prevent visual morbidity.
